# Does Transport Matter? Functional Integration of the Pollen on the Fig Wasp Body in Active and Passive Pollination of Fig Trees

**DOI:** 10.3390/plants15091305

**Published:** 2026-04-23

**Authors:** Ana Julia Peracini, Rodrigo Augusto Santinelo Pereira, Simone Pádua Teixeira

**Affiliations:** 1 Programa de Pós-Graduação em Biologia Comparada, Faculdade de Filosofia, Ciências e Letras de Ribeirão Preto, Universidade de São Paulo, Ribeirão Preto 14040-130, SP, Brazil; anajperacini@gmail.com; 2 Departamento de Biologia, Faculdade de Filosofia, Ciências e Letras de Ribeirão Preto, Universidade de São Paulo, Ribeirão Preto 14040-130, SP, Brazil; raspereira@usp.br; 3 Departamento de Ciências Farmacêuticas, Faculdade de Ciências Farmacêuticas de Ribeirão Preto, Universidade de São Paulo, Ribeirão Preto 14040-903, SP, Brazil

**Keywords:** Agaonidae, brood-site pollination, coevolution, plant–insect interactions, pollen physiology, ultrastructure

## Abstract

The obligate mutualism between *Ficus* and its pollinating wasps provides a suitable system to investigate these dynamics because it encompasses two contrasting pollination modes: active and passive. Here we compared pollen traits in an actively pollinated fig tree, *Ficus citrifolia*, and a passively pollinated species, *F. obtusiuscula*, examining pollen both at anther presentation and after deposition on the bodies of their pollinating wasps. Pollen morphology, hydration-related behavior, cytology, and reserve composition were characterized using scanning electron microscopy (conventional and modified), light and transmission electron microscopy, histochemical assays, and viability tests. Across species, pollen traits at anthesis showed broad overlap in morphology, viability and major reserve classes, indicating that these characteristics are not consistently predicted by pollination mode alone. In both species, pollen was bicellular, harmomegathic and highly viable at presentation, consistent with resilience during transport. The main divergence emerged after pollen transfer to the pollinator. In the actively pollinated species, pollen recovered from wasp thoracic pockets exhibited pronounced intracellular remodeling, including vacuolization, starch depletion, lipid redistribution and localized cytoplasmic degradation. By contrast, pollen of the passively pollinated species retained a comparatively stable cytological organization after transport despite changes in reserve distribution. These results suggest that the more pronounced cytoplasmic reorganization observed in the pollen of the actively pollinated species after deposition on the wasp body may represent a preparatory phase for rapid germination following pollination, reflecting the stronger dependence of larval development on successful flower fertilization in actively pollinated figs. More broadly, our study provides the first comparative account of pollen structural and cytophysiological dynamics on fig-wasp bodies, linking pollen cell biology to pollinator-mediated dispersal and highlighting how different pollination strategies may impose distinct selective pressures on male gametophytes.

## 1. Introduction

Pollination arises from the interplay between cellular traits of the pollen (i.e., the male gametophyte) and the ecological interactions with biotic and abiotic vectors, thereby making pollen biology a central link in plant cell biology, reproductive processes, and the evolution of floral diversity [1,2]. This connection with plant cell biology becomes particularly evident when pollen is considered as an independent plant individual composed of two or three cells, depending on the species [3,4]. Consequently, studies of pollen biology and ecology share many conceptual and methodological frameworks with investigations traditionally applied to macroorganisms (see [5,6,7]).

The conditions pollen encounters during its trajectory from dispersal to stigma reception impose selective pressures on pollen morphological and cytological traits, reflecting functional demands, including dispersal efficiency, adhesion/retention on vectors, desiccation tolerance, and germination speed [2,8]. For instance, the pollen of wind-pollinated species often exhibits unsculptured exine ornamentation and starch reserves in the vegetative cell, whereas in insect-pollinated species, they usually show elaborate exine ornamentation [9] and lipid-based reserves [10,11]. However, the correlation between starch content and wind pollination appears not to be universal [12], whereas the relationship between exine ornamentation and pollination mode may be taxon-dependent [13]. Therefore, pollen traits are shaped not only by broad pollination syndromes but also by lineage-specific mechanisms of pollen transfer and performance on stigmatic surfaces.

In this context, *Ficus* L. (Moraceae) provides a suitable system for investigating the relationship between pollen biology and pollination ecology, as fig trees are engaged in an obligate mutualism with pollinating Agaonidae wasps [14,15]. *Ficus* species exhibit two distinct pollination modes: active and passive. In active pollination, pollen grains are transported into specialized insect structures, whereas in passive pollination, pollen is loosely dispersed over the surface of the wasp’s body [16,17,18].

*Ficus* is the largest genus in Moraceae, with approximately 870 species [19], characterized by an urn-shaped inflorescence (called a fig) enclosing diclinous flowers [20]. For pollination to occur, the pollen vector must enter the enclosed inflorescence through the fig ostiole, a narrow passage formed by ostiolar bracts. Once inside, the mechanism of pollen transfer differs according to the mentioned pollination mode. In the active mode, a female agaonid wasp deliberately collects the pollen grains from the anthers and stores them in specialized structures called thoracic pockets [16,17]. After collecting the pollen, the female wasp leaves the fig and flies to another tree bearing figs with pistillate flowers in anthesis (hereafter referred to as receptive figs). Upon entering a new inflorescence, the pollinating wasp picks pollen grains from their thoracic pockets and deposits them on the stigma surfaces, while laying its eggs in the ovary of some of the pistillate flowers [21].

In passive pollination, pollen grains are naturally released from the anthers and adhere to the bodies of wasps as they move through the inflorescence [21]. The pollen transport and release in these species rely on physical processes of abdominal shrinkage and expansion in the pollinating wasp, driven by changes in ambient humidity. Galil and Neeman [18] experimentally demonstrated that upon emerging from its gall, the female of *Blastophaga psenes* Linnaeus (pollinator of *F. carica* L.) wasp has a swollen abdomen with exposed intersegmental and pleural folds due to the high humidity inside the pollen-bearing fig. After exiting the fig, the wasp’s grooming behaviour transfers part of the pollen to these pleural invaginations. Because of the lower external humidity, the abdomen contracts and the folds close, trapping the pollen grains within. When the wasp later enters a receptive fig, the higher humidity inside causes its abdomen to swell again, gradually releasing the trapped pollen from the pleural folds and facilitating pollination.

Approximately two thirds of *Ficus* species are actively pollinated [21]. Ancestral state reconstructions indicate that the ancestor of all extant fig trees was most likely actively pollinated [22]. Within the *Ficus* phylogeny, there are numerous reported cases of transitions from active to passive pollination, but not a single case in which active pollination has been regained [22]. The hypothesis that active pollination is the ancestral condition is further supported by morphological evidence, as most passively pollinating fig wasps possess vestigial thoracic pollen pockets, consistent with the hypothesis that passive pollinators are derived from actively pollinating ancestors. A notable exception is the genus *Tetrapus* Mayr, whose species pollinate the basal section *Pharmacosycea* and lack unequivocal traces of pollen pockets. Nonetheless, some *Tetrapus* species display smooth mesosomal folds bordered by setae, a structure that may represent either a morphological remnant of an ancestral pollen pocket or a preadaptation to its evolution through exaptation [20]. Active pollination may also enhance wasp development, as their larvae often feed on endosperm derived from double fertilization, likely providing a richer resource than the parthenogenetically induced tissue observed in unpollinated ovules [23,24]. Functionally, active pollination increases pollination efficiency and, as in other actively pollinated brood site mutualisms, allows a substantial reduction in pollen production [25], a feature that may contribute to the ecological success and diversification of actively pollinated *Ficus* lineages.

Despite extensive studies on the ecological and evolutionary aspects of the fig–fig wasp mutualism, the role of pollen structure and physiology during transport remains poorly understood. In particular, there is a lack of studies examining how pollen traits change between anther presentation and their deposition on the pollinator’s body, and how these changes may differ between active and passive pollination systems. This gap limits our understanding of how selective pressures during transport shape pollen performance and, ultimately, the functioning of the mutualism.

Given this context, comparative analyses of pollen morphology and physiology at anthesis and after deposition on the pollinator’s body provide a direct framework to investigate the constraints imposed during pollen transfer. Here, we test the hypothesis that contrasting pollination modes (active vs. passive) are associated with distinct pollen structural and physiological traits, reflecting different selective pressures during transport. Specifically, we aim to (i) characterize pollen morphology, cytology, and reserve composition at anthesis and after deposition on the pollinator’s body, and (ii) assess how these traits vary between actively and passively pollinated fig tree species (*Ficus citrifolia* Mill. and *F. obtusiuscula* (Miq.) Miq., respectively), providing insights into the functional integration between pollen biology and pollination mode.

## 2. Results

### 2.1. Pollen Characteristics

**Figure 1:**
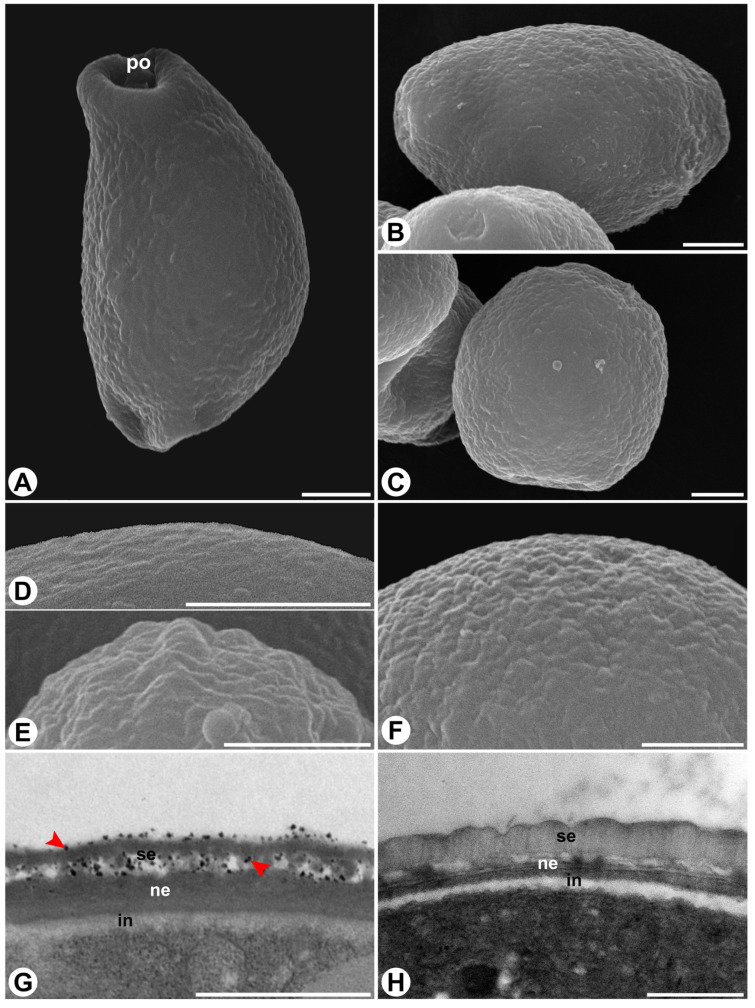
Scanning electron micrographs (**A**–**F**) and transmission electron micrographs (**G**,**H**) of pollen grains of *Ficus citrifolia* ((**A**,**D**,**E**,**G**)—left column) and *F. obtusiuscula* ((**B**,**C**,**F**,**H**)—right column). (**A**–**C**) Overview of the pollen grains. (**D**–**F**) Detail of the psilate (**D**) and fossulate (**E**,**F**) exine ornamentation. (**G**,**H**) Close-up of the pollen wall, showing the exine layers (sexine and nexine) and the intine. Note the presence of adhesive substances (arrowheads) in the pollen wall in (**G**) and their absence in (**H**). Abbreviations: in = intine; ne = nexine; po = pore; se = sexine. Scale bars: (**A**–**D**,**F**) = 2 µm; (**E**,**G**,**H**) = 1 µm.

Pollen grains in both species are dispersed as monads, very small to small in size, and isopolar, with a circular to elliptic amb. In *F. citrifolia*, pollen is predominantly oblate, occasionally suboblate or oblate-spheroidal, whereas in *F. obtusiuscula* it is mostly suboblate, sometimes oblate or spheroidal (Figure 1A–C). The mean polar diameter is slightly but significantly smaller in *F. citrifolia*, whereas no significant difference was observed in the mean equatorial diameter between the species (Table 1). Pollen viability was high in both species (~90%), with no statistically significant difference (Table 1).

**Figure 2:**
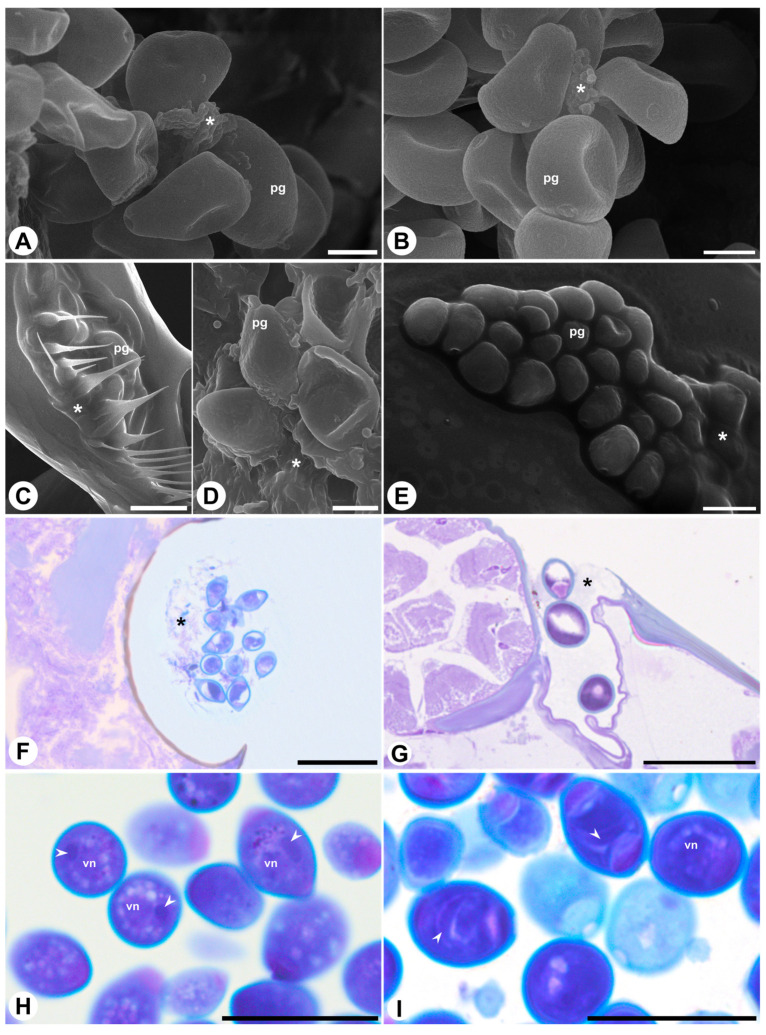
Scanning electron micrographs (modified SEM; (**A**–**E**)) and light photomicrographs (**F**–**I**) of *Ficus citrifolia* ((**A**,**C**,**D**,**F**,**H**)—left column) and *F. obtusiuscula* ((**B**,**E**,**G**,**I**)—right column) pollen. (**A**,**B**) Pollen grains in the anthers, highlighting the changes in shape displayed by the pollen grains and the adhesive substances (*). (**C**–**E**) Pollen grains in the wasps’ bodies, showing the adhesive substances (*). (**F**,**G**) Pollen in the wasp’s thoracic pockets (**F**) and body surface (**G**), stained with toluidine blue. (**H**,**I**) Pollen grains in the anthers, stained with toluidine blue, showing the nucleus of the vegetative cell and fusiform generative cell (arrowhead). Abbreviations: pg = pollen grain; vn = vegetative cell nucleus. Scale bars: (**A**,**B**,**D**) = 5 µm; (**C**,**F**–**I**) = 20 µm; (**E**) = 10 µm.

In both species, pollen grains have two, rarely three pores, without opercula or annuli. The exine is tectate with a continuous tectum in both taxa, though ornamentation and layer proportions differ. In *F. citrifolia*, the exine is psilate to fossulate, and the nexine has a similar thickness as the sexine (Figure 1D,E,G), whereas in *F. obtusiuscula* the exine is fossulate, and the sexine is thicker than the nexine (Figure 1F,H). In *F. citrifolia*, adhesive substances occur conspicuously, appearing as electron-dense amorphous material on the exine surface and within the intercolumellar spaces (Figure 1G). In contrast, in *F. obtusiuscula*, adhesive substances appear to be much less abundant, as electron-dense material was not detected on the exine surface or within the intercolumellar spaces (Figure 1H). These observations are supported by SEM (Figure 2A–E) and anatomical images (Figure 2F–I), in which adhesive substances appear as irregular, scaly, amorphous aggregates that coat and partially connect adjacent pollen grains in *F. citrifolia* (Figure 2A,C,D,F), but are less evident in *F. obtusiuscula* (Figure 2B,E,G). In both species, pollen grains are released in a bicellular state (Figure 2H,I) and are harmomegathic, as evidenced by the changes in shape observed when dehydrated (Figure 2A,B).

### 2.2. Pollen in the Anthers Versus on the Wasp’s Body

**Figure 3:**
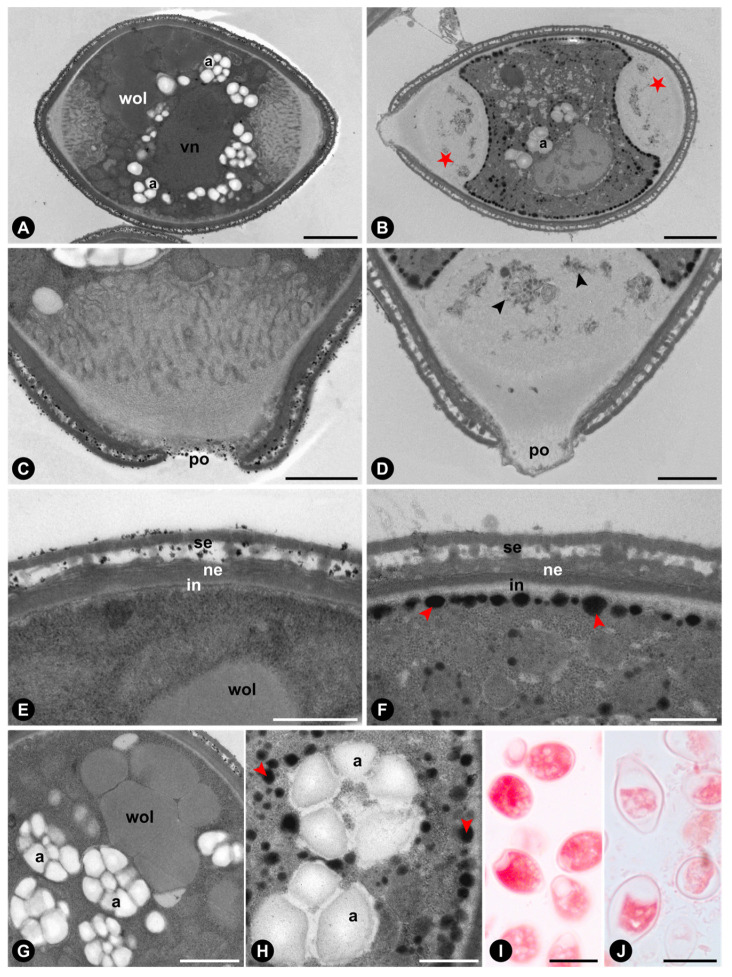
Transmission electron micrographs (**A**–**H**) and light photomicrographs (**I**,**J**) of *Ficus citrifolia* pollen from anthers (**A**,**C**,**E**,**G**,**I**) and thoracic pockets of the pollinating wasp (**B**,**D**,**F**,**H,J**). (**A**,**B**) General organization of the pollen grains, highlighting vegetative cells with differing cytoplasmic electron densities. Note osmiophilic lipid bodies adjacent to the intine, reduced starch grains, and autophagic vacuoles in the pore region (★) in (**B**). (**C**,**D**) Close-up of pollen wall and pores. Note organelle degradation within vacuoles near the pores in (**D**) (black arrowheads). (**E**,**F**) Pollen wall stratification showing the presence of adhesive substances in the intercolumellar space in (**E**) and, in contrast, their absence but the presence of osmiophilic lipid bodies (red arrowheads) near the intine in (**F**). (**G**,**H**) Lipid storage patterns in the vegetative cells, with large vacuoles containing weakly osmiophilic lipids in (**G**), whereas H exhibits numerous small osmiophilic lipid bodies (red arrowheads) surrounding the amyloplasts. (**I**,**J**) Pollen grains stained with Xylidine Ponceau for protein detection. Note the more intense red staining in (I), indicating a higher relative protein content compared to (**J**). Abbreviations: a = amyloplast; in = intine; ne = nexine; po = pore; se = sexine; vn = nucleus of the vegetative cell; wol = weakly osmiophilic lipids. Scale bars: (**A**,**B**) = 2 µm; (**C**,**D**,**G**) = 1 µm; (**E**,**F**,**H**) = 500 nm; (**I**,**J**) = 20 µm.

**Figure 4:**
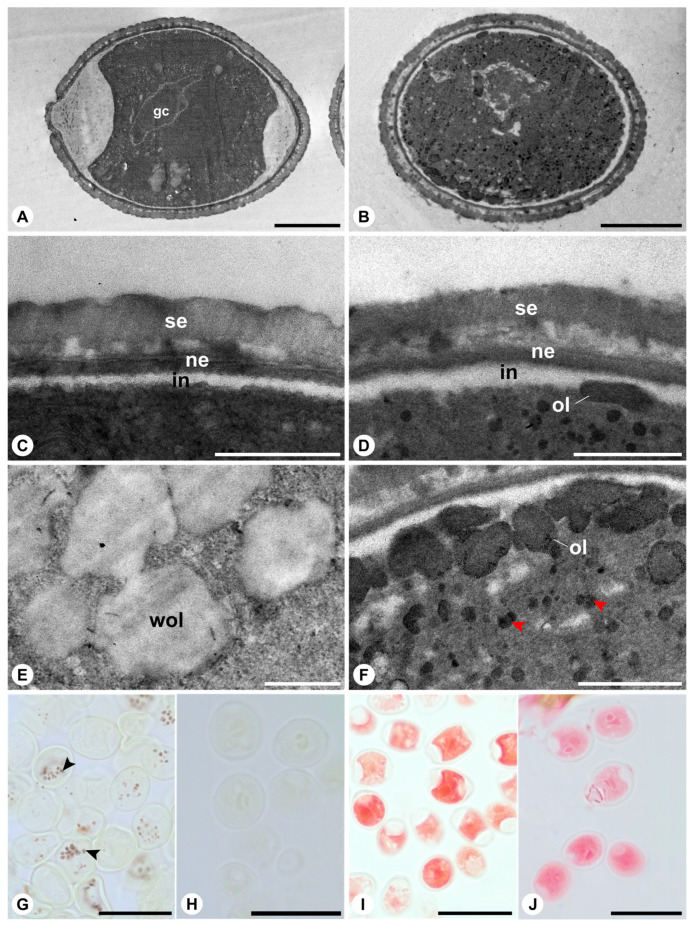
Transmission electron micrographs (**A**–**F**) and light photomicrographs (**G**–**J**) of *Ficus obtusiuscula* pollen from anthers (**A**,**C**,**E**,**G**,**I**) and on the pollinating wasp’s body (**B**,**D**,**F**,**H**,**J**). (**A**,**B**) General organization of the pollen grains, highlighting vegetative cells with dense cytoplasm. Note osmiophilic lipid bodies dispersed throughout the vegetative cell cytoplasm in (**B**). (**C**,**D**) Close-up of pollen wall. Note the absence of osmiophilic lipid bodies near the intine in (**C**) and the presence in (**D**). (**E**,**F**) Details of the vegetative cell cytoplasm, showing large vacuoles containing weakly osmiophilic lipids in (**E**), whereas (**F**) exhibits prominent osmiophilic lipid bodies. (**G**,**H**) Starch grains (arrowheads) detected by Lugol solution in (**G**), whereas they are absent in (**H**). (**I**,**J**) Pollen grains stained with Xylidine Ponceau for protein detection (red staining). Abbreviations: gc = generative cell; in = intine; ne = nexine; ol = osmiophilic lipids; se = sexine; wol = weakly osmiophilic lipids. Scale bars: (**A**) = 5 µm; B = 2 µm; (**C**,**D**,**F**) = 500 nm; (**E**) = 1 µm; (**G**–**J**) = 20 µm.

Comparisons between pollen grains from the anthers and those from the wasp’s body revealed some cytological differences (Figure 3 and Figure 4). In *F. citrifolia*, pollen grains within the anther contain a vegetative cell with numerous amyloplasts bearing large starch grains, as well as weakly osmiophilic lipids stored in vacuoles that occupy a large portion of the cell volume (Figure 3A,C,E,G), and a relatively higher protein content (Figure 3I). In contrast, pollen grains on the wasp’s body exhibit a vegetative cell with a less dense and more vacuolated cytoplasm, fewer amyloplasts (Figure 3B,D,F), autophagic vacuoles in the pore region (Figure 3B,D), prominent osmiophilic lipid bodies close to the intine (Figure 3F) and surrounding the amyloplasts (Figure 3H), and a relatively lower protein content (Figure 3J).

In *F. obtusiuscula*, both pollen grains from the anthers and those from the wasp’s body exhibit a vegetative cell with dense cytoplasm (Figure 4A,B). Pollen from the anthers contains weakly osmiophilic lipids stored in vacuoles (Figure 4C,E), amyloplasts (Figure 4G), and proteins (Figure 4I). In pollen sampled from the body of the pollinating wasp, osmiophilic lipid bodies are dispersed throughout the cytoplasm and concentrated near the intine (Figure 4B,D,F). Amyloplasts are no longer detected (Figure 4H), whereas proteins remain present in the cytoplasm. No substantial differences in Xylidine Ponceau staining intensity are observed between pollen at anther presentation and pollen recovered from the wasp body (Figure 4I,J), indicating that protein content does not vary markedly between these two conditions.

## 3. Discussion

We found no evidence that pollen morphological, morphometric, hydric, cytological, or reserve-related traits are consistently associated with pollination mode in the two *Ficus* species examined. In contrast to expectations derived from a comparative study that found that actively pollinated fig tree species have pollen grains significantly more elongated [26], the pollen of *F. citrifolia* (active pollination) and *F. obtusiuscula* (passive pollination) exhibited broadly overlapping characteristics. This pattern is consistent with evidence from species of subsection *Urostigma* [27] and sections *Americanae* and *Pharmacosycea* [28], in which no clear association was detected between pollen morphology and pollination mode. With the exception of a slightly more elongated pollen and the occasional occurrence of psilate exine ornamentation in *F. citrifolia*, both taxa share a common suite of traits. Pollen grains are predominantly 2-porate (rarely 3-porate), lack opercula and annuli, and are released in a bicellular state. In both species, grains are harmomegathic and contain starch, lipids, and proteins as reserve substances. Viability at anther presentation was high, with no significant difference between species.

The most conspicuous differences between the actively and passively pollinated species, beyond the more abundant adhesive material coating the exine of *F. citrifolia*, as predicted for actively pollinated figs [21], emerged from comparisons between pollen at anther presentation and after deposition on the pollinator’s body. In *F. citrifolia*, pollen recovered from the thoracic pockets of the wasp exhibited pronounced cytoplasmic reorganization and reserve mobilization. Specifically, these grains showed (i) the formation of large vacuoles within the vegetative cell, (ii) a reduction in the number of amyloplasts and a lower detectable protein content, (iii) a redistribution of lipids into osmiophilic oleosomes positioned near the intine, and (iv) signs of organelle degradation, particularly in regions adjacent to the pores. In contrast, pollen of *F. obtusiuscula* showed no substantial vacuolization after transfer to the wasp’s body and retained a comparatively stable cytological organization, differing mainly by the absence of detectable amyloplasts and the predominance of dispersed oleosomes within the cytoplasm. These contrasting post-dispersal responses suggest that pollen in the actively pollinated species undergoes a more pronounced structural adjustment during transport than in the passively pollinated species, with greater cytoplasmic reorganization and reserve mobilization potentially reflecting stronger selective pressures for rapid pollen germination and ovule fertilization. Experimental evidence supports this hypothesis, linking the development of actively pollinating wasps to successful ovule fertilization. In *F. citrifolia* and other actively pollinated species, figs entered by pollen-free wasps are more likely to abort, whereas larval mortality is substantially higher in retained but unpollinated figs. In contrast, in passively pollinated species, eggs are more frequently deposited in unpollinated flowers without compromising wasp offspring development [23,24,29]. Moreover, in *F. citrifolia* and four additional actively pollinated species of section *Americanae*, pollen germination occurs rapidly, often while the pollinating wasp is still alive within the receptive fig [30].

From a mechanistic cellular perspective, the cytoplasmic reorganization documented here is consistent with processes known to be essential for successful pollen germination [31,32]. In angiosperms, the initial phase of pollen germination is typically accompanied by intense autophagic activity, particularly in the vicinity of the apertures, leading to compartmentalized degradation of specific cytoplasmic regions. Experimental inhibition of autophagy results in the persistence of a cytoplasmic layer over the apertures, thereby mechanically obstructing pollen tube emergence [31]. In parallel, autophagy contributes to the polarization of lipid bodies toward the future pollen tube tip, facilitating directional growth and sustained tube elongation [32]. Notably, in phylogenetically distant taxa such as *Arabidopsis thaliana* (L.) Heynh., *Lilium longiflorum* Thunb., and *Nicotiana tabacum* L., whose pollinators do not depend on ovary fertilization for their own reproductive success, these autophagic processes are initiated only after pollen grains reach the stigma [31,32,33]. The earlier onset of cytoplasmic reorganization suggested for *F. citrifolia* may therefore represent a heterochronic shift in the timing of pollen germination, potentially driven by the tight coupling between fertilization success and pollinator fitness in actively pollinated figs.

Pollen of *Ficus* is frequently dispersed over surprisingly long distances for an insect-pollinated plant group [15]. Empirical estimates indicate that mean pollination distances can reach several tens of kilometers [34,35,36,37], and aerial plankton surveys have demonstrated that pollinators of monoecious fig trees are often transported by wind currents above the forest canopy across extensive spatial scales [38,39]. Such a dispersal strategy inevitably exposes pollen grains to marked fluctuations in temperature and, in particular, ambient humidity, potentially imposing strong selective pressures for high intrinsic viability and for mechanisms conferring tolerance to dehydration. Several features documented here are consistent with this interpretation. First, the harmomegathic behaviour observed in both species enables accommodation of cytoplasmic volume changes during dehydration–rehydration cycles [40]. Second, pollen grains remain bicellular even on the wasp body, a trait generally associated with greater longevity compared with tricellular pollen [41]. Third, the amylolysis may not only supply energy for pollen sperm cell maturation and pollen tube emergence [2,10,42] but also contribute to osmotic regulation within the vegetative cell, as starch hydrolysis helps stabilize cellular water content and thereby maintain viability under desiccating conditions [43,44]. These traits suggest that the pollen of *Ficus* is functionally adapted to withstand the particular dispersal dynamics characteristic of the fig–fig wasp mutualism.

Importantly, our study provides, to our knowledge, the first detailed comparative account of pollen structural and cytophysiological dynamics after deposition on the pollinator’s body. By moving beyond static descriptions at anther presentation, we show that pollen transport is accompanied by marked intracellular remodeling, particularly in the actively pollinated species, revealing how cytoplasmic reorganization and reserve mobilization may enhance both stress tolerance and functional readiness for germination. Collectively, these results establish a mechanistic framework linking pollen cell biology to pollinator-mediated dispersal and differential selective pressures across pollination modes, thereby advancing our understanding of how male gametophyte traits contribute to the evolutionary stability and ecological success of the fig–fig wasp mutualism. Future studies should extend this approach to *Ficus* species from other sections and further investigate the physiological changes occurring in pollen after transport by pollinating wasps.

## 4. Materials and Methods

### 4.1. Study Species and Sites

**Figure 5:**
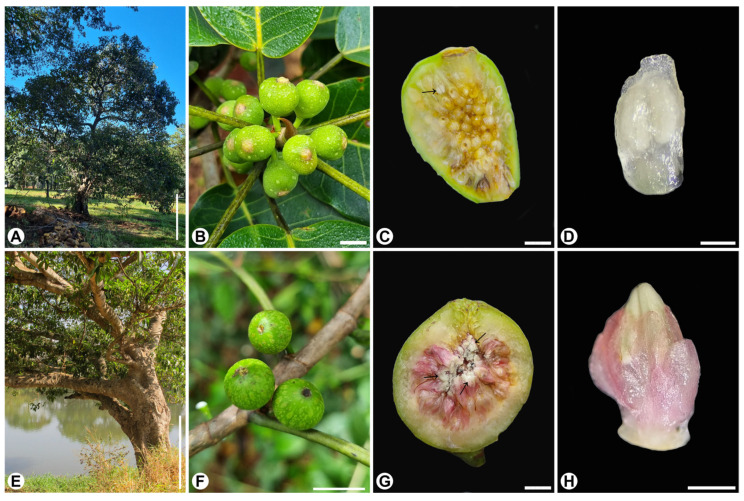
Aspects of plants and figs of *Ficus citrifolia* (**A**–**D**) and *F. obtusiuscula* (**E**–**H**). (**A**,**E**) General view of the tree. (**B**,**F**) Close-up of figs on the branch. (**C**,**G**) Internal view of the fig. Note the staminate flowers distributed throughout the fig (arrows). (**D**,**H**) Close-up of staminate flowers. Scale bars: (**A**,**E**) = 1 m; (**B**,**F**) = 1 cm; (**C**,**G**) = 5 mm; (**D**,**H**) = 1 mm.

The study was conducted between January 2023 and May 2025. *Ficus citrifolia-guaranitica* form (Figure 5A–D), section *Americanae* [45], is actively pollinated by *Pegoscapus aerumnosus* (Grandi) in the study region. It is a medium-sized, pioneer, monoecious, hemi-epiphyte species, often associated with disturbed environments. *Ficus obtusiuscula* (Figure 5E–H), section *Pharmacosycea*, is passively pollinated by an undescribed *Tetrapus* Mayr species. It is a large-sized, monoecious, freestanding species, predominantly in riparian forest [46].

Pollen of *F. citrifolia* was obtained from plants on the University of São Paulo campus in Ribeirão Preto city (21.166260° S, 47.855183° W). The campus landscape comprises gardens and lawns with a mix of native and planted tree species. Pollen of *F. obtusiuscula* was obtained from trees on the riparian forest of the Pardo River in Ribeirão Preto city, Brazil (21.100278° S, 47.760899° W).

### 4.2. Sample Collection

Figs bearing staminate flowers just before anthesis (Figure 5C,D,G,H) were collected from 10 *F. citrifolia* and 10 *F. obtusiuscula* trees. The vouchers of both species are deposited in the SPFR herbarium (FFCLRP, University of São Paulo, Ribeirão Preto, Brazil) under the records: *F. citrifolia*—SPFR 12997 and SPFR 14969; *F. obtusiuscula*—SPFR 14967. Samples of both species were also stored in 70% ethanol in the spirit collection of the Faculty of Pharmaceutical Sciences of Ribeirão Preto, University of São Paulo, under the numbers: *F. citrifolia*—621, 622; *F. obtusiuscula*—632.

Pollen grains were analyzed from two sources: (i) from anthers just before anthesis, representing the condition at the moment of pollen presentation, and (ii) from the bodies of pollinating wasps, representing the condition two hours after their emergence from the fig. Pollen grains from anthers were analyzed from approximately 20 staminate flowers from 10 figs of each *Ficus* species, while pollen grains from the wasp’s body were analyzed from 20 female wasps of *P. aerumnosus* and 20 female wasps of *Tetrapus* sp.

To obtain the pollinating wasps, between 20 and 50 figs at the stage immediately preceding wasp emergence were collected, enclosed in voile fabric bags, and maintained under laboratory conditions until natural emergence occurred. This procedure ensured that the emerging female pollinators had the opportunity to collect or become loaded with pollen under typical conditions. Approximately 2 h after emergence, the wasps were sacrificed by freezing or exposure to ethyl acetate [47], depending on the subsequent pollen analysis. The two-hour interval between emergence and processing allowed sufficient time for the wasps to perform grooming behavior and for the pollen grains to acclimate to ambient humidity and temperature outside the fig. We are aware that, under natural conditions, the time elapsed between emergence from the natal fig and entry into a fig bearing receptive pistillate flowers likely exceeds two hours. However, due to logistical constraints that limited experimental evaluation of different exposure durations, we adopted this shorter interval to capture morphological and physiological changes in pollen without the confounding effects of aging.

Samples intended for scanning electron microscopy (SEM), light microscopy (LM), and transmission electron microscopy (TEM) were fixed in Karnovsky solution (glutaraldehyde 2% and paraformaldehyde 4% in phosphate buffer, pH 7.4) [48] for 24 h and subsequently stored in 70% ethanol until processing. A subset of samples was not fixed and was instead used for modified SEM preparation, hydric behavior assays, and pollen viability tests.

Pollen was obtained according to the requirements of each analytical approach. For LM and TEM analyses, anthers and whole wasps were processed as described in the subsequent sections. For SEM analyses of pollen from anthers, CO_2_ critical-point dried or silica gel–dried samples (see Section 4.3) were gently opened, and pollen grains were dispersed onto carbon double-sided adhesive tape mounted on aluminum stubs using a fine needle; all preceding preparation steps were conducted with pollen retained within the anthers. For viability assays (Section 4.5), fresh anthers were crushed directly onto microscope slides containing a drop of diaminobenzidine solution, thereby releasing the pollen grains. Due to the extremely small size of the wasps and the limited number of pollen grains adhering to their bodies, repeated attempts to isolate pollen were unsuccessful. Consequently, in all subsequent analyses, pollen associated with pollinators was processed together with whole wasps.

### 4.3. Pollen Surface (SEM)

Staminate flowers were dehydrated in an ethanol series, CO_2_ critical-point dried in a Balzers CPD 030 dryer (Oerlikon Balzers, Balzers, Liechtenstein) and mounted on aluminum stubs. Pollen grains were exposed using a fine needle, sputter-coated with gold for 300 s in a Bal-Tec SCD 050 sputter coater (Bal-Tec, Ashford, UK), and examined using a Jeol JSM-6610LV scanning electron microscope (JEOL Ltd., Tokyo, Japan) at 25 kV. Pollen descriptive terminology followed Punt et al. [49] and Halbritter et al. [50].

In addition, pollinating wasps and staminate flowers were processed using a modified SEM preparation method. Fresh anthers and wasps were placed on filter paper inside a glass jar containing silica gel, and the jar was maintained sealed at −18 °C for 7 days, followed by an additional storage at room temperature for 10 days [51]. The samples were then mounted, sputter-coated, and observed as described above for conventional SEM. For examination of pollen on the body of *P. aerumnosus*, wasps were either positioned in ventral view to observe the thoracic pollen pockets or dissected to remove the forelegs, which were mounted laterally to expose the coxal corbicula. *Tetrapus* sp. wasps were mounted in both ventral and dorsal views on the stubs to allow observation of pollen distribution on the body surface.

### 4.4. Pollen Hydration State

To infer the hydration state of pollen (e.g., partially hydrated or partially dehydrated), we used fresh samples to compare pollen morphology before and after dehydration when subjected to SEM processing. Pollen grains fixed in Karnovsky solution were typically spherical, as revealed by conventional SEM (see Section 2). Therefore, shape changes observed after dehydration were interpreted as evidence of harmomegathic behavior, corresponding to reversible changes in pollen volume and shape associated with water loss and rehydration.

### 4.5. Pollen Viability

Pollen viability in both species was assessed using 3,3′-diaminobenzidine (DAB; 0.07% diaminobenzidine and 0.16% urea hydrogen peroxide in Tris buffer, 0.06 M), a colorimetric assay that detects peroxidase activity in the cytoplasm [52]. Fresh pollen samples were obtained from three to four flowers per fig, from three figs belonging to different individuals per species, totaling ten flower anthers per species. Each anther was gently crushed on a microscope slide in a drop of DAB solution, and 500 pollen grains were scored per slide by examining successive microscopic fields, without intentional selection bias. Grains exhibiting brown staining were considered viable, indicating cytoplasmic peroxidase activity.

### 4.6. Pollen Cytology (LM)

The fixed anther and wasp samples were dehydrated through an ethanol series, embedded in Leica Historesin, and sectioned at 3–4 µm thickness using a Leica RM 2245 rotary microtome (Leica Microsystems, Wetzlar, Germany). The sections were stained with toluidine blue O (pH 6.8) [53] to determine the number of pollen cells. Additional histochemical tests were performed to identify storage substances in the vegetative cell cytoplasm and on the pollen surface using the following reagents: Sudan Black B for lipids [54], Lugol’s solution for starch [55], periodic acid–Schiff (PAS) for polysaccharides [56], and Xylidine Ponceau for proteins [57].

### 4.7. Pollen Ultrastructure (TEM)

Karnovsky-fixed anthers and pollinating wasps were post-fixed in osmium tetroxide in 0.1 M phosphate buffer (pH 7.2) and dehydrated through an acetone series, in which the samples were submerged in 30%, 50%, 70%, 90%, 95% and 100% acetone solutions for 15 min each, at room temperature. The samples were then embedded in Araldite resin (EMS 6005) and stored at 60 °C for 72 h. Ultrathin sections (70 nm) were obtained using a Leica Reichert Ultracut S ultramicrotome (Leica Microsystems, Wetzlar, Germany) and contrasted with 2% uranyl acetate and 0.3% lead citrate for 15 min [58]. Pollen grains were examined and documented using the following transmission electron microscopes: JEOL 100CXII (JEOL Ltd., Tokyo, Japan), Tecnai Spirit (FEI Company, Hillsboro, OR, USA), and JEOL JEM-1011 (JEOL Ltd., Tokyo, Japan).

## 5. Conclusions

Our study shows that pollen traits at anthesis are broadly conserved between actively and passively pollinated *Ficus* species, indicating that pollination mode alone does not predict pollen morphology, viability, or reserve composition. In contrast, the transition from anther presentation to deposition on the pollinator’s body reveals marked divergence between pollination strategies. Pollen of the actively pollinated species undergoes pronounced cytoplasmic reorganization and reserve mobilization, whereas pollen of the passively pollinated species remains comparatively stable.

These findings suggest that the key selective pressures associated with pollination mode act during transport rather than at pollen release. The observed intracellular remodeling in actively pollinated species likely reflects functional adjustments that enhance readiness for rapid germination, consistent with the tighter coupling between fertilization success and pollinator fitness in these systems.

By linking pollen cell biology to pollinator-mediated dispersal, this study provides a mechanistic framework for understanding how contrasting pollination strategies shape male gametophyte function and contribute to the evolutionary stability of the fig–fig wasp mutualism.

## Figures and Tables

**Table 1:** Cyto-physiological characteristics of pollen from the actively pollinated *Ficus citrifolia* and the passively pollinated *F. obtusiuscula*. Quantitative variables are presented as mean ± standard deviation. *N* = number of pollen grains measured (diameter) or anthers analyzed (viability). NA = not applicable.

Traits	*N*	*F. citrifolia*	*F. obtusiuscula*	*t*-Test
Polar diameter (µm)	25	6.77 ± 0.34	7.37 ± 0.65	*t*_48_ = 4.08, *P* < 10^−3^
Equatorial diameter (µm)	25	9.12 ± 1.78	8.99 ± 0.74	*t*_48_ = 0.34, *P* = 0.74
Viability rate (%)	10	90.4 ± 7.1	91.3 ± 3.9	*t*_18_ = 0.33, *P* = 0.74
Aperture number and type	NA	2 (3) pores	2 (3) pores	NA
Number of cells (anther presentation)	NA	2	2	NA
Number of cells (wasp’s body)	NA	2	2	NA
Exine ornamentation	NA	Psilate or fossulate	Fossulate	NA
Reserve substances (anther presentation)	NA	Starch grains, lipids, proteins	Starch grains, lipids, proteins	NA
Reserve substances (wasp’s body)	NA	Less starch grains, lipids, less proteins	Lipids, proteins	NA

## Data Availability

Data are contained within the article.

## Abbreviations

The following abbreviations are used in this manuscript:

LM	light microscopy
SEM	scanning electron microscopy
TEM	transmission electron microscopy
